# Pathogenic Responses among Young Adults during the 1918 Influenza Pandemic

**DOI:** 10.3201/eid1802.102042

**Published:** 2012-02

**Authors:** G. Dennis Shanks, John F. Brundage

**Affiliations:** Australian Army Malaria Research Institute, Enoggera, Queensland, Australia (G.D. Shanks);; Armed Forces Health Surveillance Center, Silver Spring, Maryland, USA (J.F. Brundage)

**Keywords:** influenza, viruses, pandemics, 1918 influenza pandemic, cellular immunity, epidemiology, World War I, influenza virus core proteins, nucleoprotein, CD8+ lymphocytes, bacterial pneumonia, mortality

## Abstract

Of the unexplained characteristics of the 1918–19 influenza pandemic, the extreme mortality rate among young adults (W-shaped mortality curve) is the foremost. Lack of a coherent explanation of this and other epidemiologic and clinical manifestations of the pandemic contributes to uncertainty in preparing for future pandemics. Contemporaneous records suggest that immunopathologic responses were a critical determinant of the high mortality rate among young adults and other high-risk subgroups. Historical records and findings from laboratory animal studies suggest that persons who were exposed to influenza once before 1918 (e.g., A/H3Nx 1890 pandemic strain) were likely to have dysregulated, pathologic cellular immune responses to infections with the A/H1N1 1918 pandemic strain. The immunopathologic effects transiently increased susceptibility to ultimately lethal secondary bacterial pneumonia. The extreme mortality rate associated with the 1918–19 pandemic is unlikely to recur naturally. However, T-cell–mediated immunopathologic effects should be carefully monitored in developing and using universal influenza vaccines.

## Abstract 2

These responses after secondary exposures caused bacterial pneumonia and most deaths.

The influenza pandemic of 1918–19 was the most deadly single event in recorded history. Because of its unique severity and global effects, it is the prototype of a global natural disaster. In recent years, fears of recurrence of an influenza pandemic similar to that in 1918 have motivated planning, preparations, and allocations of resources by public health and other government agencies, nongovernmental organizations, medical care providers, pharmaceutical and medical device manufacturers, medical researchers, private businesses, and persons worldwide (*1*).

Because of severe consequences and current relevance of the 1918 pandemic, it is essential to review its events and effects, determine their underlying causes, and assess likelihood of a recurrence. These tasks are difficult because the 1918 pandemic occurred at the end of World War I, before influenza viruses were discovered and before influenza vaccines, antiviral and antibacterial drugs, and intensive medical care were available. Fortunately, abundant and detailed written records exist of clinical, laboratory, and epidemiologic events during the pandemic period (*2**–**6*). In addition, isolates of the virus that caused the lethal second wave of the pandemic (in the fall of 1918 in most locations) have been reconstructed from preserved remains of patients who died (*7*). These isolates have been used to determine the genetic relationships between the 1918 pandemic influenza strain and subsequent seasonal and pandemic A/H1N1 strains (*8*). Genetic relationships between the 1918 pandemic strain and strains that caused the clinically mild first wave of epidemics in 1918 and pandemics before 1918 remain undefined (*9**–**11*).

It is commonly believed that the 1918 pandemic resulted from the sudden emergence and worldwide spread of an inherently hypervirulent influenza strain. However, this view is inconsistent with several well-documented characteristics of the pandemic. In this report, we review unique, poorly understood, or unexplained clinical and epidemiologic characteristics of the 1918 pandemic. Also, we present hypotheses that are scientifically credible, consistent with the historical record, and account for epidemiologic and clinical manifestations of the pandemic. Finally, we discuss implications of our hypotheses regarding pandemic influenza preparedness and research and development of universal influenza vaccines (*12*).

## Unique and Unexplained Characteristics of the 1918 Pandemic

### Mortality and Case-Fatality Rates

Because the 1918 pandemic spread worldwide and caused unprecedented numbers of deaths, it is often presumed that the pandemic strain was unusually transmissible and that infection with the virus was inherently lethal (i.e., direct effects of the virus routinely and rapidly caused death). During the 1918 pandemic, influenza infection rates were similar to those during other pandemics of the last century; and in most affected populations, overall mortality rates were <1%, and case-fatality rates were <3% (*4**,**13*). Thus, the 1918 pandemic strain was not unusually transmissible compared with other pandemic strains (*13*); and even without definitive treatments (e.g., antiviral and antibacterial drugs) or modern life-preserving measures (e.g., mechanical ventilation, medical intensive care), most infected persons survived (*10*).

### Deaths Caused by Secondary Bacterial Pneumonia

In 1918, most pandemic-related deaths were not caused by primary influenza-related pneumonia or acute respiratory distress syndrome, and relatively few deaths occurred within the first few days after illness onset (*11*). Most deaths occurred >7 days after illness onset and were the result of secondary bacterial pneumonia caused by common colonizers of the respiratory tract, e.g., *Haemophilus influenzae*, *Streptococcus pneumoniae*, *S. pyogenes*, and *Staphylococcus aureus* (*3**–**5**,**14*). Clinical and pathologic records suggest that lethal secondary bacterial pneumonias often followed dysregulated immune responses to infections with influenza (*15**,**16*).

### Increased Mortality Rate in Young Adults (W-shaped Mortality Curve)

In general, during the 1918–19 influenza pandemic period, illness rates were highest among children of school age. However, mortality rates were highest among infants, young adults, and the elderly (Figure) (*17*). The W-shaped relationship between mortality rate and age is a unique and unexplained characteristic of the 1918 pandemic. The lack of correspondence between illness and mortality rate in relation to age belies the common views that direct pathologic effects of the virus were independently and invariably life threatening and that the usual clinical course after infection was rapid deterioration of respiratory function terminating in death.

**Figure:**
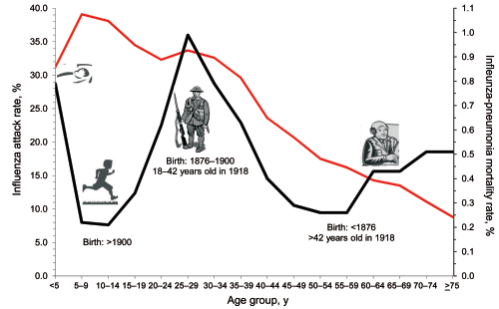
Illness attack rate (red line) and overall mortality rate (black line) for influenza-related pneumonia, by age groups of selected US populations, during the 1918 influenza pandemic period.

In 1889–90, pandemic influenza (Russian flu) spread rapidly throughout the world, and from 1890 through the winter of 1900–01, widespread epidemics of influenza-like illness recurred (*4**,**18*). The 1890–91 and subsequent epidemic waves likely were caused by variants of the 1889–90 pandemic strain (*19*). Thus, before 1918, most members of the 1875–1900 birth cohorts had been exposed to the 1889–90 pandemic influenza strain. These persons were 18–43 years old, the age groups at highest mortality risk, during the lethal second wave of the 1918 pandemic (*20*).

### Timing and Characteristics of Epidemic Waves

During 1918–19, three distinct influenza epidemic waves occurred. The first wave (mid-1918 in most locations) caused widespread illness but few deaths (3-day fever). The second wave (fall of 1918 in most locations) caused widespread illness and high mortality rates (*14*). The third wave (winter 1919) caused widespread illness but affected fewer persons and caused fewer deaths than the second wave.

The sharp contrast in the clinical expressions of infections during the first and second waves suggests that they were caused by different influenza virus strains. If the first 2 epidemic waves of the 1918–19 pandemic were caused by the same or immunologically cross-reactive influenza A (H1N1) viruses, persons affected during the first wave should have been protected from infection and, in turn, illness, secondary pneumonia, and death during the second wave. Protection from infection would have derived from neutralizing antibodies against the same or similar viral surface antigens (e.g., hemagglutinin).

There are conflicting reports regarding the immunologic susceptibility to infection during the second wave among persons who were infected during the first wave. Several reports of the experiences of localized groups (e.g., students, prisoners, military units) suggest that illness during the first wave protected from influenza during the second wave (*3**,**9*). However, our review of the medical records of all persons who served in the Australian Imperial Forces in Europe and the Middle East in 1918 documents that persons affected during the first wave were as likely to become ill, but were much less likely to die, from influenza–pneumonia during the second wave (*12*). Together, the findings suggest that infections during the first wave altered immune responses to the pandemic strain during the second wave. In turn, persons infected during the first wave had milder clinical expressions and lower mortality rates when infected with the pandemic strain during the second wave.

### Mortality Rates among Nurses and Medical Officers

During the 1918 pandemic period, military nurses and medical officers were intensively and repeatedly exposed to the influenza A (H1N1) pandemic strain in clinics, in ambulances, and on crowded open wards. However, during the lethal second wave, nurses and medical officers of the Australian Army had influenza-related illness rates similar to, but mortality rates lower than, any other occupational group (*12*). Similar observations were made in other groups of military and civilian health care workers (*21*). These findings suggest that the occupational group with the most intensive exposure to the pandemic strain had relatively low influenza-related pneumonia mortality rates during the second wave (*12*).

### Mortality Rates among Military Members with Least Service

During the fall of 1918, all 40 large mobilization/training camps throughout the United States and Puerto Rico were affected by influenza epidemics (*13**,**22*). During the camp epidemics, influenza–pneumonia mortality rates were inevitably highest among the soldiers with the least military service. In the US Army overall, 60% of those who died of influenza-related pneumonia were soldiers with <4 months of military service (*13**,**14**,**22*).

Among Australian soldiers in Europe and the Middle East in the fall of 1918, persons with the least military service also had the highest influenza-related pneumonia mortality rate (*12*). In general, in deployed Australian Army formations, soldiers were not segregated by time in military service. Thus, the nature or intensity of soldiers’ exposures to the pandemic strain likely did not vary in relation to their length of military service. During epidemics of the second wave, most soldiers were likely exposed to the same influenza A (H1N1) strain, and most of those affected were treated in the same military medical system as their counterparts regardless of seniority. Thus, the sharp differences in mortality rates in relation to length of service, in mobilization camps in the United States and deployed settings in Europe, likely reflected differences in host immune responses to the pandemic strain.

### Mortality Rates among Passengers and Crews on US Troop Transport Ships

Influenza illness rates were similar on US troop transports. However, case-fatality rates were sharply higher among soldiers who had recently congregated on the ships than among permanently assigned crewmen (*23*).

### Mortality Rates among Residents and Soldiers from Urban and Rural Areas

In the United States during the pandemic period, the influenza-related mortality rate was higher among residents of urban areas than among residents of rural areas. In contrast, the mortality rate was higher among soldiers from rural than among those from urban areas (*24*).

### Mortality Rates among Island Populations

When pandemic influenza attacked island populations, mortality rates were often high, sometimes extraordinarily so, e.g., Western Samoa (22%) and Nauru (16%) (*25**–**28*). However, some island populations had relatively low mortality rates during pandemic-related epidemics, e.g., the Philippines (1%), Puerto Rico (1%), and Hawaii (0.5%) (*25**,**29*). On other islands, mortality rates varied widely among different groups of island residents, e.g., Chamorrans (12%) versus Caroline Islanders (0.4%) on Saipan (*26*); indigenous Fijians (5.7%) versus Europeans (1.4%) on Fiji (*4*); and indigenous (Maori) (4.2%) versus European (0.5%) residents of New Zealand (*30*). Thus, on some islands, subgroups of residents who shared the same microbiological and environmental exposures had markedly different influenza-related mortality rates. Because the same influenza strain likely caused all pandemic-related island epidemics, the wide variability in mortality rates across island populations suggests that host immune factors were determinants (perhaps with other factors such as poverty, overcrowding, and malnutrition) of the clinical courses and outcomes of infections with the pandemic influenza strain.

### Clinical Expression of Infections with Similar A/H1N1 Strains in 2009 and 1918

The 1918 influenza A (H1N1) pandemic strain is genetically similar to the novel pandemic (H1N1) 2009 strain. However, clinical expressions of infections in 2009 were much less severe than in 1918, e.g., mortality rates in 1918 were >100× higher than in 2009 (*31*).

## Hypotheses

Unique and unexplained characteristics of the 1918 pandemic suggest that the risk for lethal secondary bacterial pneumonia after influenza infections depended on the nature, timing, and intensity of immune responses to the pandemic strain; and subsequently on the likelihood of exposure during transient periods of increased susceptibility to bacterial strains against which affected persons had no protective antibodies. In 1918, nearly all humans were immunologically susceptible to infection with the A/H1N1 pandemic strain; not surprisingly, the pandemic spread rapidly worldwide. The rapid spread of the pandemic with high illness attack rates in most age groups indicates that an influenza virus antigenically similar to the pandemic strain did not widely circulate among humans within at least several decades before 1918.

We hypothesize that in 1918 many persons had second lifetime exposures to an immunodominant T-cell epitope that was conserved on an internal protein of the 1918 pandemic strain and a heterosubtypic other strain (e.g., 1889 pandemic strain). When persons were reexposed to the identical epitope in 1918, epitope-specific memory CD8+ T-cells produced excessive cytokines, chemokines, immune cell activation, and epithelial cell necrosis. The immunopathologic effects of the dysregulated T-cell response transiently increased susceptibility of infected hosts to respiratory bacterial strains against which they lacked protective antibodies.

In contrast, persons who were first exposed in 1918 to the immunodominant T-cell epitope of hypothesized concern may have had primary T-cell responses that controlled virus replication without increasing susceptibility to bacterial invasion of the lower respiratory tract. Persons who had multiple prior exposures to influenza viruses and other respiratory infectious agents before 1918 had diversely partitioned memory CD8+ T-cell repertoires and extensive portfolios of bacterial strain–specific antibodies. Their immune responses to infection with the 1918 pandemic strain may have controlled virus replication without increasing their susceptibility to bacterial invasion.

Modern genetic analyses have estimated that 3 distinct variants of influenza A (H1N1) viruses co-circulated in the early 1900s (*8**,**32*). These variants were the respective prototypes of all pandemic, seasonal, and classical swine influenza A (H1N1) viruses since 1918. The first epidemic wave of the 1918 pandemic may have been the last wave of the 1889–90 Russian flu pandemic. If so, the first wave spread widely and rapidly in the face of background immunity to an influenza strain that had been circulating among humans for nearly 3 decades (*4**,**18*).

Alternatively, the first wave may have been caused by an antigenically distinct seasonal strain of influenza A (H1N1) (*8**,**32*). If so, antibodies against hemagglutinin of the seasonal strain did not provide complete protection against infection with the pandemic strain. However, because many internal proteins of human influenza viruses are conserved and strongly immunogenic (e.g., matrix 2, nucleoprotein [NP]), antibodies and memory CD8+ T lymphocytes that were produced during the first wave may have altered clinical expressions, decreased susceptibility to secondary bacterial pneumonia, and reduced deaths during the second wave (*33*).

The peak of mortality rates among young adults (W-shaped mortality curve) remains a unique and unexplained characteristic of the 1918 pandemic (*20*). Before World War I, there was relatively little global interconnectedness. Even in the most industrialized countries, many persons lived their entire lives in their birth communities and had relatively little exposure to outsiders. The situation sharply and permanently changed with the social disruptions and population dislocations precipitated by worldwide armed conflict.

Persons born before 1901 (the last year of widespread circulation of the 1890 Russian flu pandemic strain) and after 1875 (the first year after widespread epidemics of a poorly characterized influenza-like illness) were 18–43 years of age in 1918. Worldwide, members of these birth cohorts had high influenza attack rates and were likely to die from secondary bacterial pneumonia during the 1918 pandemic period (Figure).

Persons born before 1875 were >43 years of age in 1918. Before the 1918 pandemic period, they likely had been exposed to more heterosubtypes of influenza A and more respiratory bacterial strains than their younger counterparts. In general, during the pandemic period, middle-age and elderly adults had lower influenza attack rates and were less likely to die than their younger counterparts (Figure).

Also in 1918, persons born after 1901 were less likely than those older to have been exposed to the 1890 pandemic strain or displaced by war-related activities. During the pandemic period, persons <17 years of age had relatively high influenza attack rates but relatively low mortality rates (except for infants) (Figure).

We hypothesize that, soon after infection with the 1918 pandemic influenza strain, infected persons experienced a transient increase in susceptibility of the lower respiratory tract to invasion by bacteria to which they were immunologically naive. Thus, for example, those who were relatively new to their living or work environments (e.g., military recruits, soldiers on troop ships, patients on hospital wards) at the time they were infected with the 1918 pandemic strain had a relatively high risk of death from secondary bacterial pneumonia (*12*). During the course of their influenza illnesses, such persons were likely to be exposed to bacterial strains to which they lacked protective antibodies (*10*). Thus, for example, residents of rural areas were relatively unlikely to be exposed to novel strains of bacteria while recovering from influenza, and they had low pandemic-related mortality rates. In contrast, military recruits from the same rural areas were likely to be exposed to novel strains of bacteria while recovering from influenza, and they had relatively high pandemic-related mortality rates (*24*).

This interpretation explicates the somewhat counterintuitive finding that nurses, medical officers, and the crews of troop ships had high influenza attack rates but relatively low mortality rates during the lethal second wave of the 1918 pandemic. Before being infected with the pandemic influenza strain, these persons were often exposed in their occupational settings to high concentrations of diverse strains of respiratory infectious agents. Because of their extensive portfolios of respiratory bacteria strain–specific antibodies, they were naturally immune to and protected from secondary pneumonia caused by these agents (*12**,**23**,**24*).

The hypotheses presented here are consistent with the historical record and scientifically plausible. For example, studies in humans have identified an immunodominant NP-derived CD8+ T-cell epitope that is consistently presented by high frequency HLA class I molecules and recognized by cytotoxic T lymphocytes. The epitope is present on the NP of the 1918, 1976, and 2009 human pandemic strains and on most swine strains, but not on most other human strains of the past century (*34*).

Studies in pigs suggest that the NP of most swine influenza strains contains a strongly immunogenic CD8+ T-cell epitope. For example, pigs that were primed with a DNA vaccine that expresses NP, and subsequently challenged with an influenza A strain with the same NP, had dysregulated, pathogenic immune responses (*35*). Also, pigs that were primed with an inactivated swine influenza A vaccine (A/swine/Iowa/15/1930 H1N1) and subsequently challenged with a later generation swine influenza A strain with markedly different surface proteins (A/swine/Minnesota/00194/2003 H1N2) showed development of enhanced (immunologically potentiated) pneumonia that were not observed after challenge with the homologous strain (*36*). The findings have been reproduced by using pandemic (H1N1) 2009 virus as the challenge strain and adding a recombinant matrix 2 protein to the vaccine construct (*37*).

Studies in mice have documented that T-cell–mediated immunopathologic responses can contribute to severe pneumonitis when mice are exposed to a highly glycosylated influenza virus and subsequently infected with a poorly glycosylated strain. Infection with a recent seasonal influenza virus (H1N1), followed by infection with pandemic (H1N1) 2009 virus, elicited severe immunopathogenic responses (*38*). Finally, studies in ferrets have documented that those infected with pneumococci after acquiring influenza, but not before, showed development of lethal secondary pneumonia and other invasive complications (*39*).

In summary, we hypothesize that mortality risk after infection with the 1918 pandemic influenza A (H1N1) strain depended on the number, nature, and diversity of prior infections with influenza virus and respiratory bacteria. Specifically, mortality rates during the lethal second wave were highest among persons with prior exposures to heterosubtypic influenza strains that enhanced immunopathogenic effects when a person was infected with the 1918 pandemic strain and had limited exposures to other respiratory infectious agents. In such persons, infection with the pandemic strain caused high viral loads, dysregulated and pathogenic cell mediated immune responses, and transient increases in susceptibility to invasive bacterial infections. If such influenza virus–infected hosts were subsequently exposed to bacterial strains to which they had no protective antibodies, they were at high risk of acquiring life-threatening secondary bacterial pneumonia.

The unique circumstances that enabled the unprecedented mortality rates of the 1918 pandemic no longer exist on a global scale. For example, in modern times, even the most isolated communities (e.g., Pacific islanders, indigenous populations of North America, Australia, and New Zealand) are interconnected through myriad commercial and sociopolitical activities. As a result, most populations are exposed to annual seasonal influenza viruses, and most young adults are exposed to numerous viral and bacterial respiratory pathogens. Thus, compared with the situation in 1918, adults in modern communities have more diversified immune repertoires against influenza strains and bacterial respiratory pathogens. The hypotheses presented may explain at least in part the relatively low mortality rate associated with pandemic (H1N1) 2009 virus. During the 2009 pandemic, many persons who died had underlying medical conditions, including obesity, asthma, cardiovascular diseases, diabetes, and pregnancy; histopathologic changes consistent with diffuse alveolar damage; and evidence of bacterial co-infections (*40*).

Finally, the findings of this report are relevant to the research and development of a universal influenza vaccine. Candidate vaccines that contain antigens that are highly conserved across influenza A strains and strongly immunogenic must be closely monitored to ensure that T-cell–mediated immune responses to future seasonal and pandemic strains are protective but not pathogenic.
